# Profuse diarrhea after percutaneous change of a computed tomography-guided gastrostomy: endoscopic closure of a colocutaneous fistula

**DOI:** 10.1055/a-2358-1166

**Published:** 2024-07-08

**Authors:** Amani Al Khatib, Anaïs Bertrand, Henri Duboc, Olivier Bonsack, Benoit Coffin, Heithem Soliman

**Affiliations:** 1 555089 Centre de Recherche sur lʼInflammation, Inserm UMRS 1149, Université Paris Cité, Paris, France; 2 26930 Hépato-gastro-entérologie, Hôpital Louis Mourier, APHP, Colombes, France

A 71-year-old patient was admitted after the accidental dislodgement of a computed tomography (CT)-guided percutaneous gastrostomy, placed 1 year prior. The initial procedure was uneventful and facilitated rapid weight gain. A percutaneous MIC-KEY tube (Avanos, Alpharetta, Georgia, USA) was rapidly placed along the previous route. As soon as nutrition was restarted, “on and off” profuse diarrhea occurred, ceasing as the nutrition was stopped and resuming immediately upon resumption. Opacification of the MIC-KEY tube revealed a fistulous tract between the transverse colon and the skin ( Video 1 ). Conservative treatment was decided, involving the endoscopic closure of the residual colocutaneous fistula, achieved by placing an Ovesco clip (Ovesco Endoscopy, Tübingen, Germany) during a colonoscopy 1 .

Management of a silent colonic transfixation after computed tomography-guided gastrostomy. The colocutaneous fistula was revealed by diarrhea and could be safely closed with an Ovesco clip (Ovesco Endoscopy, Tübingen, Germany).Video 1

In conclusion, a transcolonic perforation may go unnoticed during the months 2 after a CT-guided percutaneous gastrostomy. Colocutaneous fistulous tract can be safely closed using an Ovesco clip during a colonoscopy 1 3 , to avoid the need for surgery.

Endoscopy_UCTN_Code_TTT_1AO_2AK

## Notes

Endoscopy E-Videos https://eref.thieme.de/e-videos
*E-Videos* is an open access online section of the journal *Endoscopy* , reporting on interesting cases and new techniques in gastroenterological endoscopy. All papers include a high-quality video and are published with a Creative Commons CC-BY license. Endoscopy E-Videos qualify for HINARI discounts and waivers and eligibility is automatically checked during the submission process. We grant 100% waivers to articles whose corresponding authors are based in Group A countries and 50% waivers to those who are based in Group B countries as classified by Research4Life (see: https://www.research4life.org/access/eligibility/ ). This section has its own submission website at https://mc.manuscriptcentral.com/e-videos .
